# Meta-Analysis of Transcriptome Data Detected New Potential Players in Response to Dioxin Exposure in Humans

**DOI:** 10.3390/ijms21217858

**Published:** 2020-10-23

**Authors:** Evgeniya Oshchepkova, Yana Sizentsova, Daniil Wiebe, Victoria Mironova, Nikolay Kolchanov

**Affiliations:** 1 Institute of Cytology and Genetics, 630090 Novosibirsk, Russia; sizentsova@bionet.nsc.ru (Y.S.); daniil.wiebe@gmail.com (D.W.); kviki@bionet.nsc.ru (V.M.); kol@bionet.nsc.ru (N.K.); 2 Faculty of Natural Sciences, Novosibirsk State University, 630090 Novosibirsk, Russia

**Keywords:** dioxin, aryl hydrocarbon receptor, meta-analysis, transcription factor binding sites

## Abstract

Dioxins are one of the most potent anthropogenic poisons, causing systemic disorders in embryonic development and pathologies in adults. The mechanism of dioxin action requires an aryl hydrocarbon receptor (AhR), but the downstream mechanisms are not yet precisely clear. Here, we performed a meta-analysis of all available transcriptome datasets taken from human cell cultures exposed to 2,3,7,8-tetrachlorodibenzo-*p*-dioxin (TCDD). Differentially expressed genes from different experiments overlapped partially, but there were a number of those genes that were systematically affected by TCDD. Some of them have been linked to toxic dioxin effects, but we also identified other attractive targets. Among the genes that were affected by TCDD, there are functionally related gene groups that suggest an interplay between retinoic acid, AhR, and Wnt signaling pathways. Next, we analyzed the upstream regions of differentially expressed genes and identified potential transcription factor (TF) binding sites overrepresented in the genes responding to TCDD. Intriguingly, the dioxin-responsive element (DRE), the binding site of AhR, was not overrepresented as much as other cis-elements were. Bioinformatics analysis of the AhR binding profile unveils potential cooperation of AhR with E2F2, CTCFL, and ZBT14 TFs in the dioxin response. We discuss the potential implication of these predictions for further dioxin studies.

## 1. Introduction

Environmental pollution by industrial emissions, waste incineration, and rocket fuel contributes to xenobiotics accumulation in the environment; these xenobiotics include a group of dioxin compounds, of which 2,3,7,8-tetrachlorodibenzo-*p*-dioxin (TCDD) is the most toxic representative [1,2,3,4]. Dioxins are one of the most potent anthropogenic poisons; in terms of overall toxicity, they exceed the most potent chemical poisons. Exposure to dioxins can damage the immune system, developing nervous system, endocrine system, and reproductive functions [4,5,6]. There is evidence that the effect of dioxins on children leads to a decrease in IQ, congenital anomalies, and weight loss in newborns [7].

The generally accepted concept of the dioxin action mechanism on the cells is that it serves as a ligand for ligand-activated transcription factor (TF) aryl hydrocarbon receptor (AhR). AhR belongs to the helix-loop-helix basic domain PER-ARNT-SIM (bHLH/PAS) subfamily and regulates the expression of a large number of genes via dioxin-responsive elements (DREs), also known as xenobiotic-responsive elements (XREs), and has the consensus 5′-TNGCGTG-3′ [8]. In the inactivated state, AhR is located in the cytoplasm in a complex with the dimeric chaperone 90 kDa heat shock protein (Hsp90), as well as with the AhR-interacting protein (AIP) and with the p23 co-chaperone [9,10]. After binding to dioxin, the AhR/Hsp90 complex translocates to the cell nucleus, where it decomposes, and AhR (with c p23) forms an active transcriptional complex with the aryl hydrocarbon receptor nuclear translocator (ARNT) [11]. ARNT also belongs to the bHLH family and directly binds to DRE [12]. In vivo DNA footprinting showed that AhR binds to the 5′-CACGCNA/T-3′, and ARNT with 5′-GTG-3′ [13]. The molecular mechanisms underlying the action of dioxin on the cell are still poorly understood. The key transcription factor mediating the effects of dioxin is known, but the exact mechanisms at the cellular level are not yet clear and require further research.

To study the cellular response mechanisms to dioxin compounds, we performed a meta-analysis of all available RNA-seq and microarray human datasets obtained under the action of TCDD. The aim of the study was to identify the TCDD targets that were systematically affected over multiple datasets. This helped us to predict novel or poorly studied mediators of the dioxin response.

## 2. Results

### 2.1. Identification of TCDD Targets in Humans

We analyzed all available whole-genome datasets obtained under the action of TCDD on different human cell lines. The individual microarray and RNA-Seq datasets were preprocessed according to similar protocols to obtain the lists of differentially expressed genes (DEGs) (see Section 4.1 and Section 4.2). As TCDD mildly affected transcription of the genes, we set a mild criterion for false discovery rate (FDR < 0.2). Twenty datasets that under this criterion yielded more than 100 up- and down-regulated DEGs were taken for further meta-analysis (Table 1). PCA analysis of these datasets showed a nice clustering of the transcriptional responses detected in different studies, with a few very specific responses (Figure S1). Gene ontology (GO) annotation of all individual datasets showed that they are informative enough with relevant GO terms significantly overrepresented (e.g., “immune system process”, “inflammatory response”, “anatomical structure morphogenesis”, etc.).

**Figure 1:**
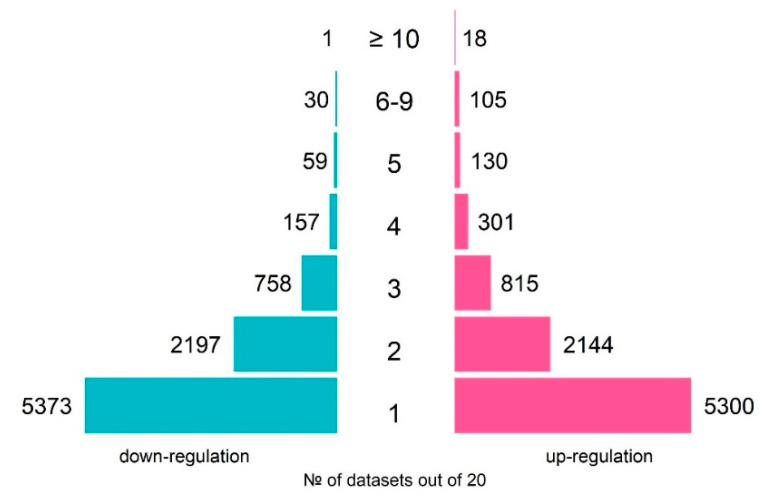
An overview of the numbers of TCDD-responsive genes over 20 analyzed transcriptomes on human cell lines: number of up-regulated genes (pink rectangles) and number of down-regulated genes (blue rectangles).

The total number of genes, which were differentially expressed in response to TCDD in at least one of the datasets, was 8813 of up-regulated and 8575 of down-regulated genes (Figure 1). As the experimental design significantly varied between the experiments, the DEG lists overlapped partially. However, many genes changed their expression in response to TCDD systematically. For example, *CYP1A1* and *CYP1B1*, known as coding enzymes involved in the metabolism of xenobiotics and associated with the TCDD response [23], were activated in 15 and 16 of the 20 analyzed datasets, respectively (Table 2).

### 2.2. Robust TCDD Targets: Known Knowns and Known Unknowns

First, we looked at the genes that responded to TCDD in the most robust way in half of the tested datasets (10 out of 20). There were 19 such genes, and some of them are known to be associated with the dioxin response or the AhR pathway (Table 2); for the remaining genes, no associations were found in the literature (Table 3). TCDD inducible poly(ADP-ribose) polymerase (*TIPARP*), encoding a negative regulator of AhR [25], was induced the most robustly—in 17 out of 20 datasets. AhR repressor (*AhRR*) is another robust TCDD target that provides for negative feedback—in 15 out of 20 datasets [27].

**Figure 2:**
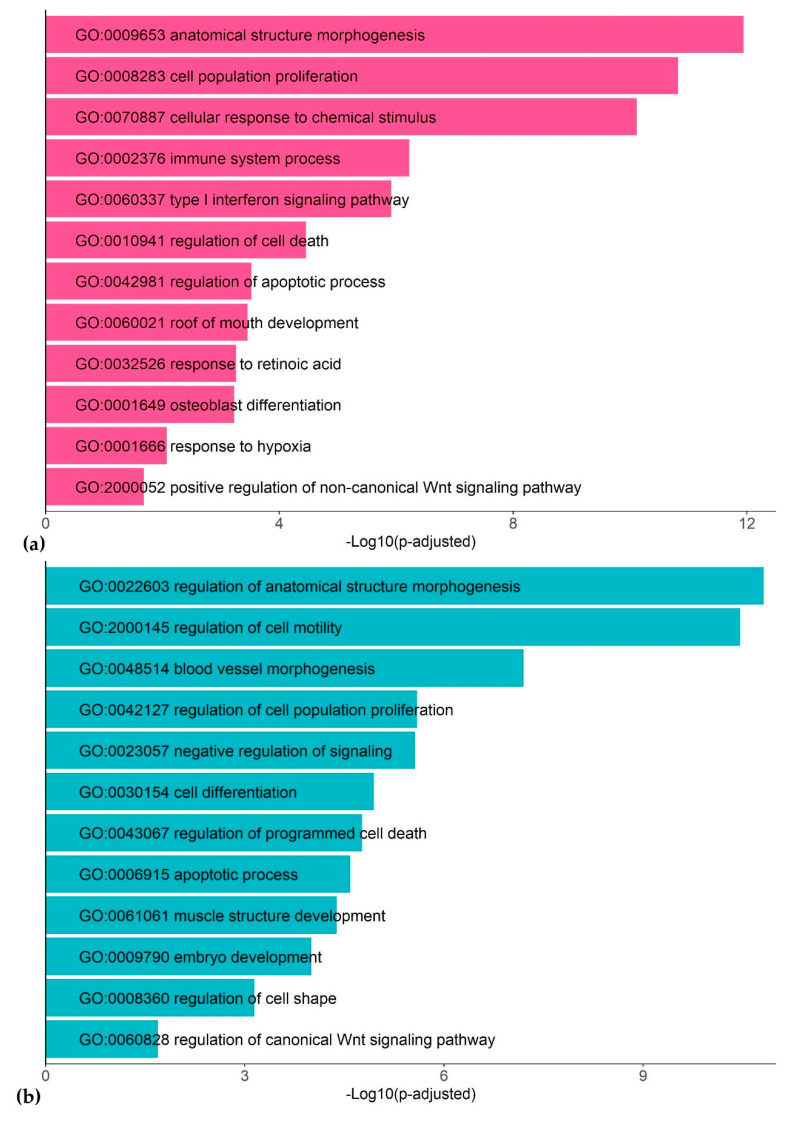
Representative gene ontology (GO) terms associated with TCDD response in human cell lines: up-regulated genes (**a**) and down-regulated genes (**b**).

Functional annotation of the DEGs frequently encountered in different datasets (at least in 4 out of 20 datasets) detected many functionally related groups, highlighting the complexity of the dioxin response in humans (Figure 2; Tables S1 and S2). We discuss the most representative functional groups and involved genes below.

Skeletal morphogenesis. There are many TCDD targets associated with skeletal morphogenesis. *DKK1*, coding a negative regulator of bone development, is the only down-regulated gene among the most robust TCDD targets (down-regulated in 11 of 20 tested datasets) (Table 2). Another robust TCDD target *RUNX2* encodes the transcription factor that regulates bone development. Other genes systematically induced by TCDD and implicated in skeletal morphogenesis are *FOSL2*, *SALL4*, *MSX2*, *ADAM12*, *BST2*, and *FAM20C* (Table S1). Previously, they were almost never discussed in relation to dioxin.

Apoptosis, cell proliferation, and cancerogenesis. There is a large set of genes involved in essential cell processes, such as cell proliferation and apoptosis, that are either up-regulated (e.g., *FZD7*, *MYC*, *CSK*, *ABCG2*, *GDF15*, *CABLES1*) or down-regulated (e.g., *CPA4*, *PHLDA1*, *ANKRD1*, *CRIP2*, *FN1*) (Table S1). Robust TCDD target *BMF* (up-regulated in 8 of 20 analyzed RNA-seq datasets), is involved in apoptosis induction [33]. *RUNX1*, encoding the transcription factor that is involved in hematopoietic stem cell proliferation [34], is robustly activated by TCDD (in 11 of 20 datasets, Table 3). Both proliferation and apoptosis programs are largely affected by TCDD, this may explain the association of dioxin with cancerogenesis processes. The relationship between dioxin compounds and cancerogenesis is debated in the Discussion.

Immune system regulators and inflammation response. TCDD robustly activates many regulators of the immune system associated with particular host defense processes. The most robust responses detected by the meta-analysis were for *SECTM1*, *IL1R1*, *CEBPD*, *LACC1*, *GADD45A*, *OAS1*, *C1S*, *COLEC12*, *GBP2*, *IER3*, and *GALNT10*.

Retinoic acid. Three of the most robust TCDD targets participate in the metabolism of all-trans-retinoic acid (RA), a vitamin A derivative and an essential morphogen involved in embryonic development [35]: *ALDH1A3*, *CYP1A1*, *CYP1B1* (Table 2) [36,37,38]. Functional annotation analysis (Figure 2) highlights that there are many other TCDD targets from this metabolic pathway, e.g., *LRAT* and *DHRS3* (Table S1). We consider in more detail the crosstalk between RA and TCDD in the Discussion.

### 2.3. Search for Cis-Elements Associated with Dioxin Response

To identify possible mediators of the dioxin response in addition to AhR/ARNT, we studied if there are any other DNA motifs systematically overrepresented in the upstream regions of the TCDD-regulated genes. We performed both unsupervised and supervised searches.

#### 2.3.1. Unsupervised Search: No Predetermined Gene Lists and TF Binding Sites

**Figure 3:**
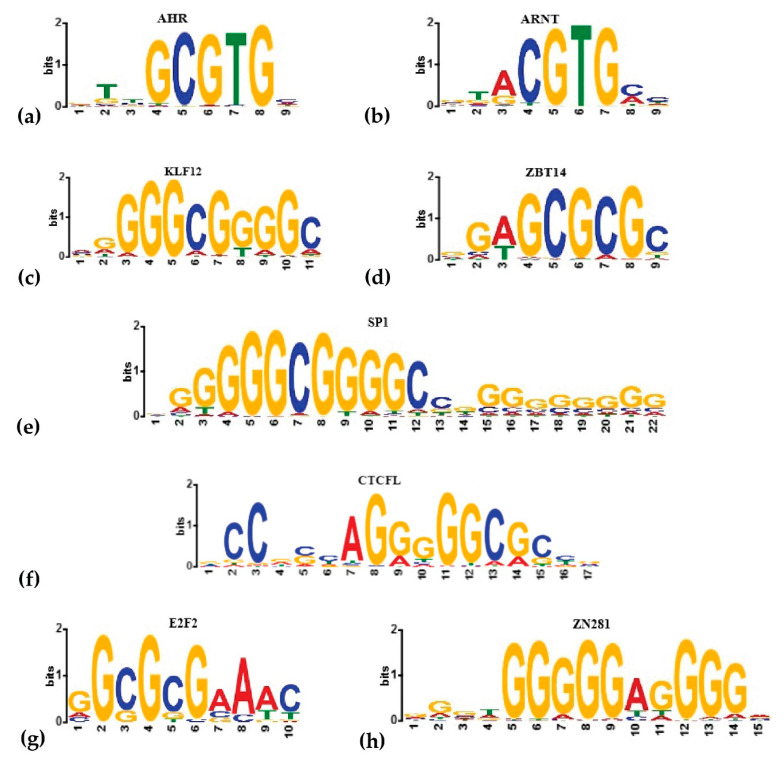
The binding sites of known (**a**,**b**) and predicted by MetaRE or HOMER (**c**–**h**) transcriptional regulators of the transcriptional response to dioxin. The logos were generated in the Hocomoco database [40].

For the unsupervised search, we applied MetaRE [39] software packages to all datasets on TCDD exposure (see Materials and Methods). In each dataset, we looked for the k-mers that are overrepresented in the upstream regions of induced or repressed gene sets. The analysis was done with different lengths of upstream regions, but here we discuss the results taken for (–1500; +1), as the most representative. MetaRE detected hundreds of motifs frequently encountered in dioxin-regulated promoters under the stringent threshold for *p* value < 1 × 10^−16^ (Figure S2). Known dioxin-responsive elements (DRE; consensus GCGTG) were not the most overrepresented among identified k-mers. For hepta- and octamers, the motifs containing GCGTG were significant only under a very mild threshold, behind hundreds of more significant motifs (the rank of GCGTG/CACGC in the list of detected potential cis-elements are shown in Table S3). DRE was noticeable under a stringent threshold as a pentamer for activated or repressed by TCDD genes and as a hexamer only for TCDD-repressed genes (Table S3). The most significantly overrepresented k-mers for those both induced and repressed by TCDD was the group of GC-rich motifs that did not match classic DRE consensus (Table S4). All octamers were GC-rich, with only 9% containing one A/T. We used the TOMTOM tool to analyze the overrepresented octamers and found significant matches with known TF binding sites. Most of the GC-rich octamers were recognized as the potential binding sites for specificity protein (Sp1) and Krüppel-like factor 12 (KLF12) (Table S4, Figure 3).

Previously, an alternative AhR-binding site was shown, which the reporting authors called a non-consensus XRE (NC-XRE). This site contains repeats of the 5′-GGGA-3′ tetramer [41]. AhR was shown to recruit Krüppel-like factor 6 (KLF6), form the heterodimeric complex AhR/KLF6, and bind to NC-XRE [42]. We also found four octamers that harbor a 5′-GGGA-3′ tetramer. TOMTOM identified them as potential binding sites of SP1, KLF12, Zinc finger protein 281 (ZN281), and CCCTC-binding factor (CTCFL) (Table S5).

#### 2.3.2. Supervised Search: Robust Dioxin-Responsive Genes and Known TF Binding Sites

In an alternative search, we analyzed promoters of dioxin targets to identify overrepresented known TF binding sites. For that, we applied the HOMER tool with the Hocomoco database of TF binding sites [40] as the reference. HOMER recognized (FDR < 0.05) binding sites of 134 and 126 TFs overrepresented in upstream regions of dioxin-activated or dioxin-repressed genes, respectively (Table S6). Among them, there are many GC-rich sites, including the binding sites for nine members of the KLF family, Sp1–Sp4 group, Zinc finger and BTB domain-containing protein 14 (ZBT14), ZNF281, and others (Figure 3, Table S6). HOMER detected non-GC-rich sites as well, particularly a set of TFs from the E2F family (E2F1–E2F7) (Figure 3). It is noteworthy that the binding sites of known dioxin response regulators were again not highly represented, with AhR sites ranked 60th and 113th and ARNT binding sites ranked 64th and 91st in up- and down-regulated by dioxin genes, respectively.

Together, supervised and unsupervised searches for cis-elements overrepresented in dioxin-regulated promoters gave us a wide spectrum of potential regulators of the dioxin-response.

### 2.4. Composite AhR-Binding Elements Analysis

As many motifs unrelated to DRE were found to be enriched in dioxin-responsive promoters, we asked whether some of them are the binding sites for AhR partners. To test this hypothesis, we studied if AhR binding sites co-occur with the motifs of AhR’s potential interactors and form composite elements (CE) of a specific structure. For that, we applied the MCOT Toolbox [43] to the AhR binding profile [22] with the AhR-binding site as an anchor and a Hocomoco collection for the partner motif candidates. MCOT detected 43 TFs that were significantly overrepresented in AhR profile CEs, all of them with full or partial overlaps of the binding sites (Table S7). Among them, the classical CE of the dioxin response, AhR-ARNT, was detected in the maximal number of peaks (27% of peaks, *p* value < 1 × 10^−15^). In this CE, directly oriented AhR and ARNT binding sites overlap by 8nt (Figure S3). Another example, AhR-ZBT14 CEs with inverted orientation and a partially overlap by 6nt of the binding sites, was present in 1.4% of peaks (*p* value < 1 × 10^−15^). ZBT14 has not been considered as a potential AhR co-regulator previously, however, our study suggests it as a candidate AhR partner protein.

## 3. Discussion

### 3.1. Meta-Analysis of Transcriptomes

**Figure 4:**
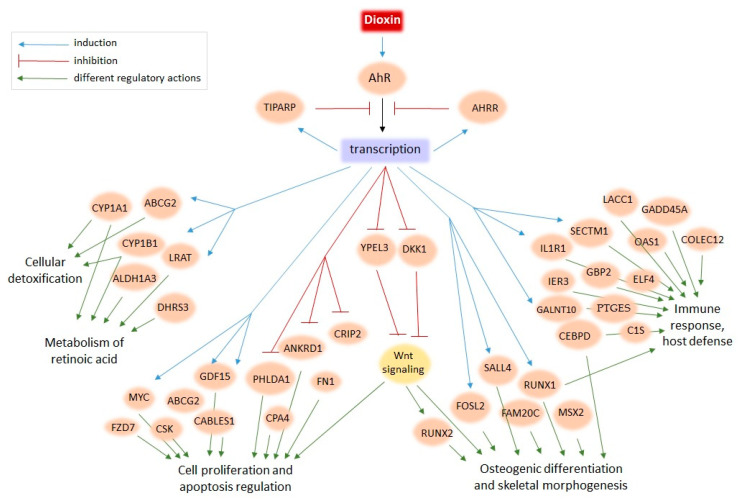
The model of dioxin action on human cells based on the present meta-analysis of transcriptome data.

We performed a meta-analysis of human transcriptomes exposed to TCDD to find new candidate genes of the dioxin response. Although the designs of whole-genome experiments on TCDD treatment varied from study to study—with different cell lines, TCDD dosage, time of treatment, and even expression profiling technologies—we still were able to detect the common signatures (Figure 1 and Figure 3). For example, *TIPARP*, encoding a negative regulator of the dioxin response, was induced in the majority of whole-genome studies, as were *CYP1A1*, *CYP1B1*, and *ALDH1A3*, well-known markers of cellular response to dioxin (Table 2). In this study, we extended the repertoire of robustly regulated by dioxin genes and highlighted the molecular trends in the dioxin response. Figure 2 and Figure 4 summarize this information. We discuss some functionally-related dioxin-responsive groups below.

Immune response. Exposure of an organism to xenobiotics, in particular dioxin, is a challenge for the immune system, therefore, it is not surprising that the biggest group of genes robustly induced by TCDD relate to the host defense processes. Of the most robust TCDD targets, *SECTM1* was associated with immune system response [44], but it was not well-characterized. Some well-studied genes related to the immune system and systematically affected by TCDD include *IL1R1*, encoding IL-1 receptor type 1, and *CEBPD*, encoding a transcription factor regulating immune and inflammatory responses, both differentially expressed in 8 of 20 tested datasets (Table S1). *PTGES* (differentially expressed in 5 of 20 datasets), a mediator of prostaglandin synthesis that takes part in inflammation response, has been shown to be activated by the AhR signaling pathway [45]. Many other genes that are associated with immune processes and systematically responded to TCDD have not yet been studied in relation to dioxin, e.g., *LACC1*, *GADD45A*, *OAS1*, *C1S*, *COLEC12*, *GBP2*, *IER3*, and *GALNT10* (Table S1). Although a number of candidate genes responding to dioxin and related to the immune system processes were found, a meta-analysis of more specific data, e.g., on immune system cells, is needed to clarify the immune response to TCDD.

RA metabolism. Another functional gene group systematically affected by TCDD relates to RA metabolism (*ALDH1A3*, *LRAT*, *CYP1A1*, *CYP1B1*, and *DHRS3*). Indeed, RA metabolism is compromised under dioxin exposure [26,46], however, the crosstalk between RA and TCDD is not evident [47]. Most of the identified robust targets have been discussed in the literature before in relation to dioxin, except *DHRS3*. Vitamin A metabolizes in two ways: it is either esterified by LRAT and stored or is reversibly oxidized to retinaldehyde by retinol dehydrogenases (DHRS3, in particular), and further irreversibly oxidized to RA [48]. It was shown previously that RA excess induces cleft palate as does TCDD exposure, and TCDD effects depend on RA signaling [49]. Our study predicted DHRS3 and LRAT could be essential mediators of TCDD action on embryonic development via the RA pathway.

Skeletal morphogenesis. A notable group of the robust dioxin-responsive genes mediates bone development and anatomical morphogenesis (Figure 2 and Figure 4). Among the known dioxin targets, there is *DKK1*, encoding an inhibitor of Wnt signaling [50], that was systematically down-regulated by TCDD (Table 2). There is evidence that RA up-regulates *DKK1* and *DKK2,* inactivating the Wnt signaling pathway [51]. Thus, the TCDD–DKK–Wnt relationship is further evidence of the interplay of the AhR and RA pathways. Another important regulator of skeletal morphogenesis, in particular osteoblastic differentiation, is a downstream target of the Wnt signaling pathway, RUNX2 (Table 2) [52]. Note that according to the meta-analysis, *RUNX2* expression was significantly increased in 14 out of 20 (and down-regulated in 1 out of 20) RNA-seq datasets. However, in a number of published works, it was shown that the expression of this gene is decreased under the TCDD action [29,53]. We assume that the contradiction in the experimental data is due to the dysregulation of the *RUNX2* gene: Wnt signaling activates it, while dioxin-induced AhR signaling down-regulates Wnt signaling and causes developmental defects [54]. Other genes robustly induced by TCDD are implicated in bone tissue maturation: *FOSL2*, *SALL4*, *MSX2*, *ADAM12*, *BST2*, and *FAM20C*. Multiple TCDD targets within the same gene network may explain the TCDD effects on craniofacial skeleton development [55] and imply that TCDD affects master regulators of bone development.

Cell proliferation, apoptosis, cancerogenesis. Wnt-signaling also contributes to the regulation of cell proliferation, apoptosis, and cancerogenesis. One of the members of this regulatory chain is protein yippee-like 3 (*YPEL3*), which is down-regulated in 7 of 20 analyzed datasets (Table S1). YPEL3 suppresses the Wnt/β-catenin signaling and its further regulation of downstream genes [56]. Another promoter of cancerogenesis that was identified by our meta-analysis as a robust dioxin target is the transcription factor RUNX1 (Table 3). *RUNX1* up-regulation has previously been shown after exposure to cytotoxic agents; upon overexpression, RUNX1 reduced proliferation, promoted apoptosis, and augmented the DNA damage response in bone marrow cells [34]. Interesting, *RUNX1* also has relatively high expression in the thymus (SRP056969 in SRA). In addition, we identified other robustly down-regulated genes that participate in the regulation of proliferation, apoptosis, and cancerogenesis, including *FN1*, *PHLDA1*, *ANKRD1*, *CPA4*, *CRIP2*, *MYC*, *FZD7*, *CSK*, *CABLES1*, *ABCG2*, *GDF15,* and others (Table S1, Figure 4). Thus, our analysis of genes robustly activated by TCDD supports the idea that dioxin does not induce cancerogenesis per se, but dysregulates cell proliferation and apoptosis, as well as the immune system, which could cause cancer.

### 3.2. Transcriptional Regulation

Another approach we used in the study was the analysis of cis-regulatory regions of dioxin-responding genes. Surprisingly, the well-known dioxin responsive element was not overrepresented; instead, many GC-rich motifs were detected as being highly enriched in promoters of dioxin-responsive genes. To study what these motifs are, we compared them with known TF binding sites and analyzed AhR-binding regions in more detail. In Table 4, we summarize the information derived by MetaRE, Homer, and MCOT tools for the most prominent potential regulators of the dioxin response, and we discuss some of these potential regulators below. Namely, we detected E2F2 and ZBT14 as potent AhR co-regulators in the dioxin response, with ZBT14 predicted in every search performed. In addition, Sp1, KLF12, and ZNF281 were detected by MetaRE and HOMER, but not MCOT. Thus, they still might be major regulators of the dioxin response, but probably without heterodimerization with AhR on DNA.

Sp/KLF regulators. Different members of the Sp/KLF family were among the top matches detected by all applied algorithms. The family consists of Sp1–9 and Sp1-related KLF1–18 that have highly conserved DNA binding domains recognizing GC-rich sequences (GGGGCGGGG and GGTGTGGGG) [57]. Among them, two TFs were predicted the most robustly: Sp1 and KLF12 (Table 4). Sp1 is a known mediator of the dioxin response: This TF activates *AhR* transcription [58], and AhR recruits Sp1 to regulate *CYP1A1* [59]. Transcription factors Sp1 and Sp3 also enhance *AhRR* [60]. This gene encodes a protein that binds to AhR ligands and DNA binding sites and suppresses the effect of activated AhR. Thus, Sp1/Sp3 negatively regulate AhR-mediated cell response through AhRR activation. There is also a link between the dioxin response and KLF6–AhR in the non-canonical signaling pathway where it forms a heterodimeric DNA-binding complex with KLF6 for participation in cell cycle regulation [61]. We also predicted that KLF12 is involved in the dioxin response, although there is no evidence in the literature. Therefore, it can be a prospective target to study further, as KLF12 is an essential regulator of embryo development, affecting the attachment of the embryo to the endometrial epithelium through the regulation of the leukemia inhibitory factor (*Lif*) gene [62]. KLF12 also plays a role in cancerogenesis and cell proliferation [63,64].

ZNF281 transcription factor. ZNF281 binding sites were overrepresented in dioxin-responsive and AhR binding regions. ZNF281 is a Krüppel-type zinc-finger transcriptional regulator with elevated expression levels in the placenta, adult kidney, liver, and lymphocytes [65]. It has not been studied in relation to dioxin thus far, but its role in the regulation of molecular and physiological processes suggest ZNF281 as a prospective target. It mediates DNA reparation processes [66], cell proliferation, migration, invasion, and metastasis of colorectal cancer by inhibiting the Wnt/β-catenin pathway [67], regulates neuronal differentiation [68], and induces the inflammatory response [69].

ZBT14 transcription factor. ZBT14 binding sites are not only enriched with dioxin-responsive genes, but also with a notable number of AhR binding peaks (AhR–ZNF281 CE) with an overlap (Table S7), suggesting that ZBT14 cooperates with AhR in the regulation of some genes. ZBT14, also known as ZFP161, ZNF478, and ZF5 in mice, is ubiquitously expressed and known to regulate many essential regulators such as *c-myc*, *X-mental retardation 1* (*FMR1*) gene, *Klf9*, *Foxp1*, and others [70,71,72,73,74]. In a breast cancer study, it was demonstrated that a decreased *ZFP161* level was associated with a poor clinical forecast [75]. It was also postulated that ZBT14 is a player in some metabolic and inflammation processes because it is involved in the regulation of *Interleukin-6* (*Il6*) and *Lif* in skeletal muscle [76].

E2F2 transcription factor. E2F2 binding sites were also found to be associated with the AhR-mediated dioxin response (Table 4). E2F2 participates in the regulation of cell proliferation and post-injury tissue repairs [77]. However, it was also shown to be associated with cancerogenesis, e.g., nasopharyngeal carcinoma, colon cancer, and others [78,79]. The link between this TF with the dioxin response and AhR signaling has been shown previously [80]. E2F2 negatively regulated the AhR pathway in T-lymphocytes after TCDD exposure. Here, we suggest the mechanism of this regulation: through CE of specific structures (Table S7) for which E2F2 and AhR either compete or cooperate with each other.

Transcriptional repressor CTCFL. CTCFL (or CCCTC-binding factor or BORIS (brother of the regulator of imprinted sites)) regulates testis-specific expression in spermatogenesis and is known to be a cancer antigen [81]. Recently it was supposed that CTCFL promotes regulatory chromatin interactions and therefore associates with cancerogenesis [82]. Here, we suggest that CTCFL can compete or cooperate with AhR on CE of particular dioxin-responsive genes.

### 3.3. Future Research

In this work, we focused on the robust responses and set aside the specific responses detected under particular conditions. The datasets used for the meta-analysis were obtained at different concentrations (from 1 to 100 nM), durations of TCDD exposure (from 6 h to 3 weeks), and cell cultures (cancer, embryonic, liver-derived lines, etc.) (Table 1). Identifying the specific responses, e.g., cell-type-specific, is an important task, but it requires more whole-genome datasets being available for meta-analysis. For example, when comparing the transcriptional responses in different studies, we see that most of the transcriptomes cluster together, but there are some specific responses (Figure S1). We cannot study specific responses through meta-analysis until we have more of such cases. Although many genes were highlighted as associated with the response to dioxin, many of them may be involved not in the primary, but the secondary response to TCDD. AhR can trigger a cascade of responses, inducing the functioning of a number of other transcription factors that directly regulate the transcription of identified robust genes. In addition, as we identified many known regulators of developmental processes, cell cycle, and differentiation as TCDD-responsive genes, their response to dioxin can partially explain the harmful effects of dioxin on the developing nervous, endocrine, and reproductive systems.

Here, we also predicted a number of TFs involved in the transcriptional response to dioxin (Table 4). Identified TFs can act independently or, as was proposed in this work, through heterodimerization or competition with AhR. Table 4 suggests that there might be three modes of AhR action in the TCDD response: (1) direct from the DRE located close to the transcription start site; (2) cooperative with other TFs whose binding sites are located in the upstream regions or link the distal enhancers with the transcription start site; and (3) indirect via AhR targets or AhR-independent regulators. All these questions require further experimental studies to understand the dioxin response and the ways to prevent its harmful effects.

## 4. Materials and Methods

### 4.1. Datasets

We searched all publicly available transcriptome studies on dioxin action on humans in Gene Expression Omnibus (GEO) using keywords “dioxin” and “human” and found twelve series of microarray datasets (GSE7765, GSE14553, GSE16160, GSE24193, GSE34249, GSE35034, GSE46874, GSE69844, GSE69845, GSE69849, GSE69850, GSE122518) and seven series of RNA-Seq datasets (GSE63935, GSE80953, GSE83886, GSE98515, GSE122002, GSE114552, GSE141711). After processing the datasets (see Section 4.2) we selected 9 microarrays (from 5 series) and 11 RNA-Seq (from 6 series) datasets. The processed ChIP-Seq dataset for AhR GSE90550 was taken from GEO. Additional details are given in Table 1.

### 4.2. Data Processing and Identification of Dioxin-Responsive Genes

We used R software version 4.0.2 and Bioconductor version 3.11 to process transcriptomics data. In the first step, we processed each individual dataset independently. The raw expression data were normalized with RMA and TMM algorithms for microarray and RNA-seq datasets, respectively [83,84]. To identify DEGs between control and TCDD-treated samples, we used the package *limma* [85] for microarray and *edgeR* [84] for RNA-seq datasets. The significance of expression changes in the microarray data were estimated by t-test and in the RNA-seq data by quasi-likelihood F-test. The raw *p* values were corrected by the Benjamini–Hochberg multiple testing procedure to adjust the FDR. FDR ≤ 0.2 was set as the criterion for DEGs irrespective of to the fold-change level. Datasets in which the number of activated or repressed DEGs were greater than 100 were included in the further meta-analysis (Table 1).

In the second step, we compared the lists of DEGs to identify the robust TCDD targets. The number of experiments in which the gene must be detected as DEG to be included in the list of systemically induced genes was equal to four experiments (*p* value = 0.016). The threshold was estimated from a binomial distribution with the probability of the gene being DEG by chance equal to 0.05.

### 4.3. Functional Annotation of Dioxin-Responsive Genes

Functional annotation was made using the topGO R package with default settings, and the Fisher exact test was used for significance estimation. Annotations between genes and GO terms were retrieved from org.Hs.eg.db R package.

### 4.4. Motif Discovery in Promoters and Peaks of Dioxin-Responsive Genes

R package MetaRE [39] was used to identify overrepresented cis-elements in upstream regions. Upstream regions of 19,815 *Homo sapiens* genes (those with expression unambiguously detected by the microarrays) were taken from GENCODE Release v27. We performed the study for (−500; +1), (1000; +1), (−1500; +1), and (−2000; +1) lengths of upstream regions. As the results were comparable with a higher overrepresentation of classic DREs in (−1500; +1) regions, here we discuss the results only for the latter.

As the foreground, we used upstream regions of genes that significantly changed their expression in response to TCDD, and as the background, the upstream regions of genes that did not change their expression. MetaRE output sequences were compared with known transcription factor binding motifs from the HOCOMOCO v11 core database (http://hocomoco11.autosome.ru/ [40] with the TOMTOM tool [86] using the euclidean distance. The hits with an E-value < 0.05 were considered as significant matches.

HOMER v4.10 known motif enrichment [87] was performed on upstream regions. The search of known motifs was performed against the HOCOMOCO v11 core motif library. Up- and down-regulated dioxin-responsive genes were analyzed separately.

### 4.5. The Search of Potential AhR Interacting Partners

We used the MCOT tool [43] to identify pairwise TF interactions in AhR-binding regions. As the anchor motif, we took the AhR position-weighted matrix (PWM) from the HOCOMOCO collection. PWMs for the rest of the TFs from this collection were screened for potential partner motifs that co-occurred with AhR. The spacer length for CE was taken between 0 and 29. The significance of CE enrichment was estimated by the adjusted *p* value < 1 × 10^−15^.

## Figures and Tables

**Table 1:** Summary on the datasets for the studies of 2,3,7,8-tetrachlorodibenzo-*p*-dioxin (TCDD) action on the human cell cultures used in the meta-analysis. Only datasets that passed the quality control are presented. GEO: Gene Expression Omnibus.

№	GEO Accession Number	Library Strategy	Platform	Time and Concentration of TCDD Treatment	Cell Culture	Replicates	References
1	GSE46874	Microarray	Affymetrix Human Gene 1.0 ST Array	30 h, 25 nM	HepaRG cells	3	[14]
2	GSE69845	Microarray	Affymetrix Human Genome 219U Array	6 h, 1 nM	MCF-7 breast cancer cells	3	[15]
3				6 h, 10 nM	MCF-7 breast cancer cells	3	
4				6 h, 100 nM	MCF-7 breast cancer cells	3	
5	GSE69849	Microarray	Affymetrix Human Genome 219U Array	6 h, 1 nM	Ishikawa cells	3	[15]
6				6 h, 10 nM	Ishikawa cells	3	
7				6 h, 100 nM	Ishikawa cells	3	
8	GSE69850	Microarray	Affymetrix Human Genome 219U Array	6 h, 100 nM	HepaRG	3	[15]
9	GSE122518	Microarray	Illumina HumanHT-12 v4.0 expression beadchip	24 h, 1 nM	HepaRG cells	4	[16]
10	GSE63935	RNA-Seq	Illumina HiSeq 2500	48 h, 3 nM	The mixture of cells: combined neural progenitor cells, endothelial cells, mesenchymal stem cells, and microglia/macrophage precursor	2	[17]
11				144 h, 3 nM		2	
12	GSE98515	RNA-Seq	Illumina HiSeq 1500	6 h, 10 nM	MCF-7 breast cancer cells	4	[18]
13				6 h, 100 nM	MCF-7 breast cancer cells	4	
14	GSE122002	RNA-Seq	Illumina NextSeq 500	96 h, 2 nM	Mel1 embryonic stem cells (before differentiation)	2	[19]
15				96 h, 2 nM	Mel1 embryonic stem cells (2-days after of differentiation)	2	
16	GSE83886	RNA-Seq	Illumina HiSeq 2500	504 h, 2 nM	BEAS-2B bronchial epithelial cell	2	[20]
17				504 h, 10 nM	BEAS-2B bronchial epithelial cell	2	
18	GSE114552	RNA-Seq	Illumina Genome Analyzer II	24 h, 10 nM	CRL-4003 decidual stromal cells	3	-
19				144 h, 10 nM	CRL-4003 decidual stromal cells	2	
20	GSE141711	RNA-Seq	Illumina HiSeq 2500	24 h, 1 nM	HepG2 liver hepatocellular carcinoma cell	5	[21]
21	GSE90550	ChIP-Seq	Illumina HiSeq 2500	24 h, 10 nM	MCF-7 breast cancer cells	1	[22]

**Table 2:** The most robustly affected genes in response to TCDD that are known to be associated with either the dioxin response or the AhR pathway. The gene function annotations were taken from the UniProtKB [24].

Gene Name	Full Name	Functions	TCDD Response in 20 Datasets		Association with AhR Pathway
			Up-Regulation	Down-Regulation	
*TIPARP*	TCDD inducible poly(ADP-ribose) polymerase	Acts as a negative regulator of AhR by mediating mono-ADP-ribosylation of AhR	17	0	[25]
*CYP1B1*	Cytochrome P450 family 1 subfamily B member 1	Oxidizes a variety of structurally unrelated compounds, including steroids, fatty acids, retinoid and xenobiotics	16	0	[23]
*ALDH1A3*	Aldehyde dehydrogenase 1 family member A3	Required for the biosynthesis of retinoic acid in the embryonic ocular and nasal regions	15	0	[26]
*CYP1A1*	Cytochrome P450 family 1 subfamily A member 1	Oxidizes a variety of structurally unrelated compounds, including steroids, fatty acids, and xenobiotics	15	0	[23]
*AhRR*	Aryl-hydrocarbon receptor repressor	Mediates dioxin toxicity and is involved in regulation of cell growth and differentiation. Represses the transcription activity of AhR	15	0	[27]
*NFE2L2 (NRF2)*	Nuclear factor erythroid 2-related factor 2	Transcription factor that plays a key role in the response to oxidative stress: binds to antioxidant response elements present in the promoter region of many cytoprotective genes, and promotes their expression, thereby neutralizing reactive electrophiles	15	0	[28]
*RUNX2*	Runt related transcription factor 2	Transcription factor involved in osteoblastic differentiation and skeletal morphogenesis	14	1	[29]
*SLC7A5 (LAT1)*	Large neutral amino acids transporter small subunit 1; L-type amino acid transporter 1	The heterodimer with SLC3A2 functions as a sodium-independent, high-affinity transporter that mediates uptake of large neutral amino acids such as phenylalanine, tyrosine, L-DOPA, leucine, histidine, methionine, and tryptophan	14	1	[18]
*DKK1*	Dickkopf-1	Locally inhibits Wnt-regulated developmental processes such as limb development, somitogenesis, and eye formation. In adults, Dkk1 is implicated in bone formation and bone disease, cancer, and Alzheimer disease	1	11	[30]

**Table 3:** The most robustly affected genes in response to TCDD that were not previously known to be associated with the AhR pathway. The gene function annotations were taken from the UniProtKB [24]. All genes are robustly up-regulated.

Gene Name	Full Name	Functions	TCDD Response in 20 Datasets	
			Up-Regulation	Down-Regulation
*IER3*	Radiation-inducible immediate-early gene IEX-1	May play a role in the ERK signaling pathway by inhibiting the dephosphorylation of ERK. Acts also as an ERK downstream effector mediating survival	13	0
*SECTM1*	Secreted and transmembrane protein 1	May be involved in thymocyte signaling	12	0
*PHLDA1*	Pleckstrin homology-like domain family A member 1	Seems to be involved in the regulation of apoptosis	12	0
*SLC7A11 (xCT)*	Cystine/glutamate transporter	Sodium-independent, high-affinity exchange of anionic amino acids with high specificity for an anionic form of cysteine and glutamate	12	1
*RUNX1*	Runt-related transcription factor 1	Forms the heterodimeric complex core-binding factor (CBF) with CBFB. The heterodimers bind to the core site of a number of enhancers and promoters, including murine leukemia virus, polyomavirus enhancer, T-cell receptor enhancers, etc. Essential for hematopoiesis	11	1
*EDC3*	Enhancer of mRNA-decapping protein 3	Binds single-stranded RNA. Involved in the process of mRNA degradation and in the positive regulation of mRNA decapping	11	0
*TPRA1*	Transmembrane protein adipocyte-associated 1	Regulates early mouse embryogenesis [31]	11	0
*TTC39C*	Tetratricopeptide repeat protein 39C	Appears to be necessary for proper MAP Kinase and Hedgehog signal transduction in developing muscle cells, as well as muscle cell differentiation [32]	11	0
*VIPR1*	Vasoactive intestinal polypeptide receptor 1	A receptor for VIP. The activity of this receptor is mediated by G proteins that activate adenylyl cyclase	10	0
*GAD1*	Glutamate decarboxylase 1	Catalyzes the production of GABA	10	1

**Table 4:** Summary table for known and predicted transcriptional regulators of dioxin transcriptional response. Binding sites of these transcription factors (TFs) were found to be significantly overrepresented in promoters and AhR peaks of TCDD-responsive genes. Three tools were used in the analysis: (1) MetaRE for an unsupervised search over multiple datasets of dioxin-regulated genes (stringent criterion was applied *p* < 1 × 10^−16^); (2) HOMER for the search of known TF binding sites within promoters of genes that are robustly regulated by dioxin (FDR < 0.05); (3) MCOT for the search of potential TF partners to AhR based on the co-occurrence of binding sites (*p* value < 1 × 10^−15^).

TF Binding Sites	Upstream Regions of Dioxin−Regulated Genes		Composite Elements in AhR-Binding Regions
	MetaRE	HOMER	MCOT
AhR	+ *	+	+
ARNT	−	+	+
E2F2	−	+	+
KLF12	+	+	−
CTCFL	+	+	−
Sp1	+	+	−
ZBT14	+	+	+
ZNF281	+	+	−

* found as an overrepresented pentamer in up- and down-regulated genes and as overrepresented hexamers in down-regulated genes.

## Supplementary Materials

Supplementary Materials can be found at https://www.mdpi.com/1422-0067/21/21/7858/s1.

## Abbreviations

ABCG2	ATP-binding cassette sub-family G member 2
ADAM12	Disintegrin and metalloproteinase domain-containing protein 12
AhR	Aryl hydrocarbon receptor
AhRR	Aryl-hydrocarbon receptor repressor
AIP	AhR-interacting protein
ALDH1A3	Aldehyde dehydrogenase 1 family member A3
ANKRD1	Ankyrin repeat domain-containing protein 1
ARNT	Aryl hydrocarbon receptor nuclear translocator
BMF	Bcl-2-modifying factor
BST2	Bone marrow stromal antigen 2
C1S	Complement C1s subcomponent
CA9	Carbonic anhydrase 9
CABLES1	CDK5 and ABL1 enzyme substrate 1
CE	Composite element
CEBPD	CCAAT/enhancer-binding protein delta
COLEC12	Collectin-12
CPA4	Carboxypeptidase A4
CRIP2	Cysteine-rich protein 2
CSK	Tyrosine-protein kinase CSK
CTCFL	CCCTC-binding factor
CYP1A1	Cytochrome P450 family 1 subfamily A member 1
CYP1B1	Cytochrome P450 family 1 subfamily B member 1
DEG	Differentially expressed genes
DHRS3	Short-chain dehydrogenase/reductase 3
DKK1	Dickkopf-1
E2F2	Transcription factor E2F2
FAM20C	Extracellular serine/threonine protein kinase FAM20C
FDR	False discovery rate
FN1	Fibronectin
FOSL2	Fos-related antigen 2
FZD7	Frizzled-7
GADD45A	Growth arrest and DNA damage-inducible protein GADD45 alpha
GALNT10	Polypeptide N-acetylgalactosaminyltransferase 10
GBP2	Guanylate-binding protein 2
GDF15	Growth/differentiation factor 15
GEO	Gene Expression Omnibus
GO	Gene Ontology
Hsp90	90 kDa heat shock protein
IER3	Radiation-inducible immediate-early gene IEX-1
IL1R1	Interleukin-1 receptor type 1
KLF	Krüppel-like factor
LACC1	Laccase domain-containing protein 1
Lif	Leukemia inhibitory factor
LRAT	Lecithin retinol acyltransferase
MSX2	Homeobox protein MSX-2
MYC	Myc proto-oncogene protein
NC-XRE	Non-consensus XRE
NDRG1	N-myc downstream-regulated gene 1 protein
OAS1	2′-5′-oligoadenylate synthase 1
PHLDA1	Pleckstrin homology-like domain family A member 1
PTGES	Prostaglandin E synthase
PWM	Position-weighted matrix
RA	All-trans-retinoic acid
RUNX1	Runt-related transcription factor 1
RUNX2	Runt related transcription factor 2
SALL4	Sal-like protein 4
SECTM1	Secreted and transmembrane protein 1
Sp1	Specificity protein 1
TCDD	2,3,7,8-tetrachlorodibenzo-*p*-dioxin
TIPARP	TCDD inducible poly(ADP-ribose) polymerase
TF	Transcription factor
XRE	Xenobiotic-responsive elements
YPEL3	Protein yippee-like 3
ZN281	Zinc finger protein 281
ZBT14	Zinc finger and BTB domain-containing protein 14
