# Supplementary material for: Meta-Analysis of Transcriptome Data Detected New Potential Players in Response to Dioxin Exposure in Humans
Source: Int J Mol Sci. 2020 Oct 23;21(21):7858. doi: 10.3390/ijms21217858 (PMC7672605; doi:10.3390/ijms21217858)
Supplement: Supplementary file 1 [file ijms-21-07858-s001.zip › Supplementary Figure S2.pptx]

## Slide 1
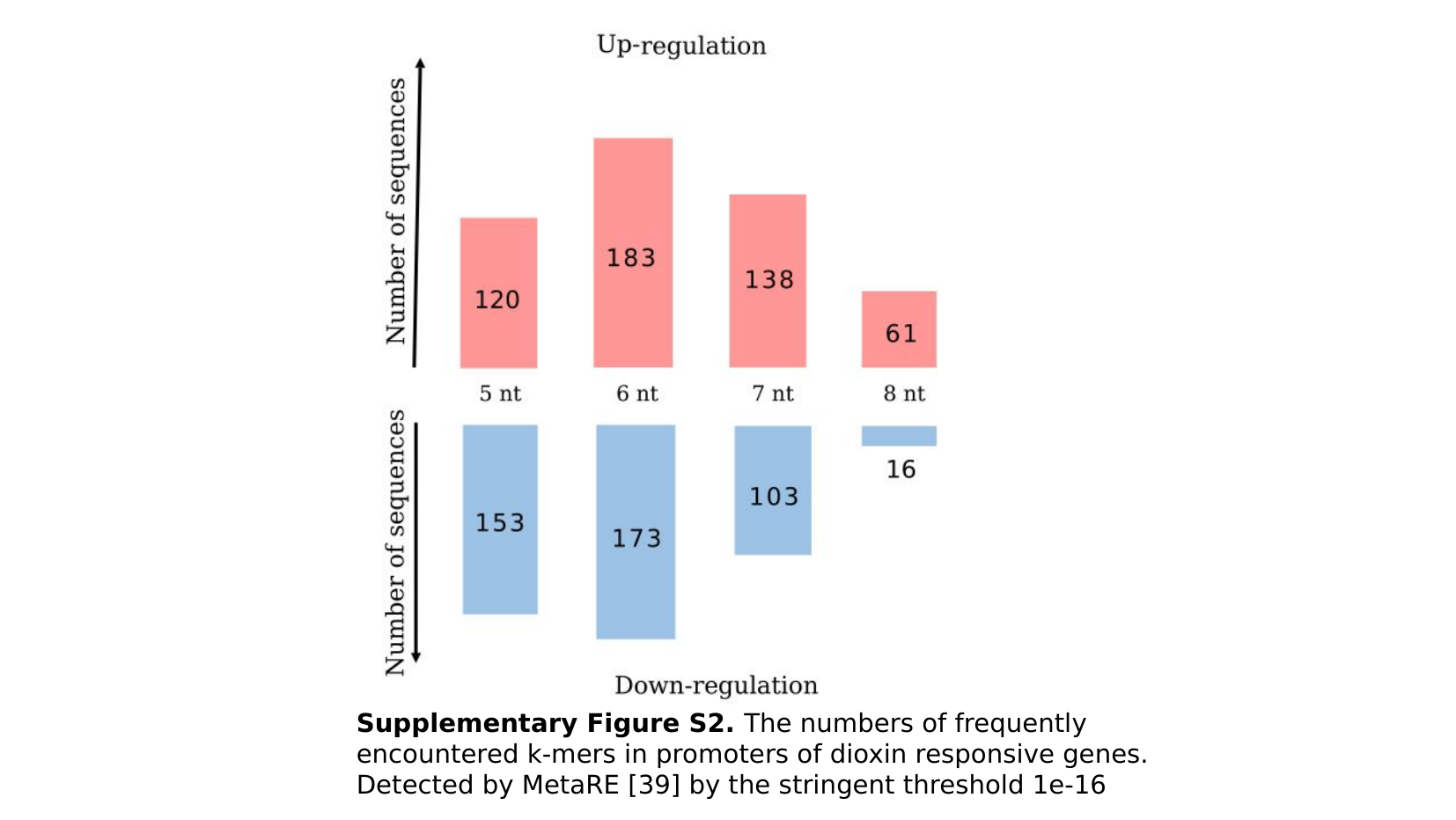

Supplementary Figure S2. The numbers of frequently encountered k-mers in promoters of dioxin responsive genes.
Detected by MetaRE [39] by the stringent threshold 1e-16
