# Supplementary material for: Meta-Analysis of Transcriptome Data Detected New Potential Players in Response to Dioxin Exposure in Humans
Source: Int J Mol Sci. 2020 Oct 23;21(21):7858. doi: 10.3390/ijms21217858 (PMC7672605; doi:10.3390/ijms21217858)
Supplement: Supplementary file 1 [file ijms-21-07858-s001.zip › Supplementary Figure S3.pptx]

## Slide 1
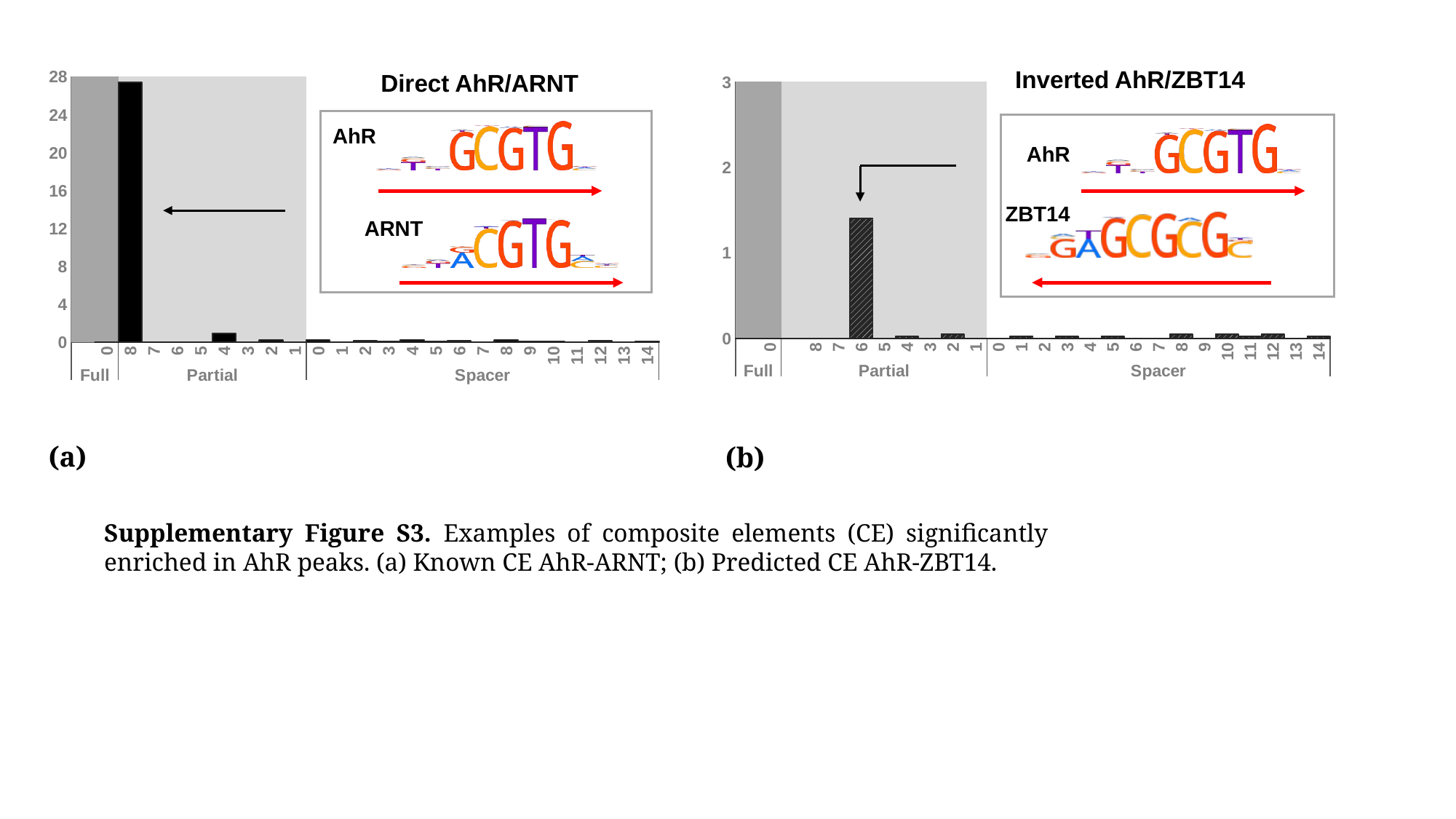

### Chart
| Category | | | DirectAP |
|---|---|---|---|
| | 28.0 | None | None |
| 0 | 28.0 | None | 0.0 |
| 8 | None | 28.0 | 27.43295 |
| 7 | None | 28.0 | 0.0 |
| 6 | None | 28.0 | 0.0 |
| 5 | None | 28.0 | 0.0 |
| 4 | None | 28.0 | 0.91954 |
| 3 | None | 28.0 | 0.025543 |
| 2 | None | 28.0 | 0.255428 |
| 1 | None | 28.0 | 0.0 |
| 0 | None | None | 0.280971 |
| 1 | None | None | 0.051086 |
| 2 | None | None | 0.178799 |
| 3 | None | None | 0.076628 |
| 4 | None | None | 0.280971 |
| 5 | None | None | 0.127714 |
| 6 | None | None | 0.153257 |
| 7 | None | None | 0.051086 |
| 8 | None | None | 0.280971 |
| 9 | None | None | 0.076628 |
| 10 | None | None | 0.127714 |
| 11 | None | None | 0.051086 |
| 12 | None | None | 0.153257 |
| 13 | None | None | 0.051086 |
| 14 | None | None | 0.076628 |Inverted AhR/ZBT14
Direct AhR/ARNT
### Chart
| Category | | | Invert |
|---|---|---|---|
| | 6.0 | None | 0.0 |
| 0 | 6.0 | None | 0.0 |
| | None | 6.0 | 0.0 |
| 8 | None | 6.0 | 0.0 |
| 7 | None | 6.0 | 0.0 |
| 6 | None | 6.0 | 1.404853 |
| 5 | None | 6.0 | 0.0 |
| 4 | None | 6.0 | 0.025543 |
| 3 | None | 6.0 | 0.0 |
| 2 | None | 6.0 | 0.051086 |
| 1 | None | 6.0 | 0.0 |
| 0 | None | None | 0.0 |
| 1 | None | None | 0.025543 |
| 2 | None | None | 0.0 |
| 3 | None | None | 0.025543 |
| 4 | None | None | 0.0 |
| 5 | None | None | 0.025543 |
| 6 | None | None | 0.0 |
| 7 | None | None | 0.0 |
| 8 | None | None | 0.051086 |
| 9 | None | None | 0.0 |
| 10 | None | None | 0.051086 |
| 11 | None | None | 0.025543 |
| 12 | None | None | 0.051086 |
| 13 | None | None | 0.0 |
| 14 | None | None | 0.025543 |
AhR
AhR
ZBT14
ARNT
(a)
(b)
Supplementary Figure S3. Examples of composite elements (CE) significantly enriched in AhR peaks. (a) Known CE AhR-ARNT; (b) Predicted CE AhR-ZBT14.
