# Supplementary material for: Meta-Analysis of Transcriptome Data Detected New Potential Players in Response to Dioxin Exposure in Humans
Source: Int J Mol Sci. 2020 Oct 23;21(21):7858. doi: 10.3390/ijms21217858 (PMC7672605; doi:10.3390/ijms21217858)
Supplement: Supplementary file 1 [file ijms-21-07858-s001.zip › Supplementary Table S3.docx]

**Supplementary Table 3.** Position numbers of the nearest dioxin-responsive element consensus (GCGTG or CACGC) among found potential cis-elements in regulatory region of up-regulated genes (**Up-**) and regulatory region of down-regulated genes (**Down-**).

|  | **Up-** | **Down-** |
| --- | --- | --- |
| **Pentamers** | 106 | 84 |
| **Hexamers** | 203 | 111 |
| **Heptamers** | 304 | 175 |
| **Octamers** | 345 | 60 |
