# Supplementary material for: Meta-Analysis of Transcriptome Data Detected New Potential Players in Response to Dioxin Exposure in Humans
Source: Int J Mol Sci. 2020 Oct 23;21(21):7858. doi: 10.3390/ijms21217858 (PMC7672605; doi:10.3390/ijms21217858)
Supplement: Supplementary file 1 [file ijms-21-07858-s001.zip › Supplementary Table S5.docx]

**Supplementary Table 5.** MetaRE octamer search results - the frequently encountered in dioxin-responsive upstream regions octamers similar to NC-XRE. Matches to known TF binding sites were found by TOMTOM [88].

| **№** | **Sequence** | **Meta P-Value** | **Tomtom**  **matches**  **(E-value < 0.05)** |
| --- | --- | --- | --- |
| **Up-regulation** | | | |
| 1 | AGGGGGCG | 1.12E-20 | CTCFL, INSM1, SP1, KLF9 |
| 2 | CCTCCCCC | 1.44E-18 | ZN281, WT1, ZSC22 |
| 3 | GGGAGGGG | 4.65E-18 | ZN281, ZN467, KLF12, WT1, SP1, KLF5 |
| 4 | GCGGGGAC | 1.10E-17 | n.s. |
| 5 | GGCGGGGA | 4.34E-17 | KLF12, SP1 |
